# The effect of dynamic and static norm messages on physical activity behaviours: Evidence from two experimental studies

**DOI:** 10.1111/aphw.70209

**Published:** 2026-08-19

**Authors:** Mark Stevens, Holly Freeman, Clare Newton, Gabrielle Guillermo‐Tregoning

**Affiliations:** ^1^ School of Medicine and Psychology The Australian National University Canberra Australian Capital Territory Australia; ^2^ School of Psychological Sciences Monash University Melbourne Victoria Australia

**Keywords:** exercise, health behaviour, motivation, self‐efficacy, social influence

## Abstract

Evidence suggests that dynamic norm messages, which communicate a positive collective change in behaviour over time, are more effective for changing some behaviours than messages that highlight current normative behaviour (i.e. static norm messages). However, it is unclear whether this is true for physical activity behaviours. We sought to advance understanding by testing this in two pre‐registered experiments with mixed designs. In Study 1 (*N* = 105), we assessed the effects of the two types of messages on young adults' performance (distance travelled) and exertion (maximum and average heart rate) during a cycling task. In Study 2 (*N* = 163), we assessed their effects on older adults' physical activity interest, intentions and self‐reported weekly activity. Static norm messages consistently had stronger effects on behaviour than dynamic norm messages, leading to greater improvements in exercise task performance and total weekly physical activity. There was also some evidence that both types of messages boosted physical activity outcomes indirectly via their positive impact on motivation and self‐efficacy. Findings suggest that communicating current norms in a way that either provides people with a ‘target’ to aim for or that speaks to the proportion of others that are outdoing them can be particularly effective for improving physical activity behaviours.

## INTRODUCTION

Norms are a prominent feature of the social environment and can meaningfully impact people's behaviours, including their health‐related behaviours (see Kim et al., 2019; Reynolds et al., 2015, for reviews). Although this impact can be negative (e.g. encourage drinking and drug taking; Stevens et al., 2021), considerable research has shown that norms can also be harnessed to improve behaviour. This research has typically used *static* norm messages that highlight *current* normative behaviours (e.g. Labrie et al., 2013; Priebe & Spink, 2015). More recently, researchers have tested the benefits of *dynamic* norm messages that communicate a positive collective *change* in behaviour over time, finding some evidence that these can have stronger effects than static norm messages (e.g. Loschelder et al., 2019; Sparkman & Walton, 2017).

However, it is unclear whether dynamic norm messages are more effective for changing *physical activity behaviour*—a complex behaviour to change given its wide array of determinants and continuing, multifaceted and effortful nature (e.g. see Schüler et al., 2025). We sought to test this and, in the process, generate important applied knowledge. Global self‐report data indicate that 31.3% of adults do not meet physical activity guidelines (Strain et al., 2024). Indeed, although lower rates of insufficient activity have been reported in some countries (e.g. 25.5% in Australia and 19.0% in the United Kingdom, the two countries in which we collected data; Strain et al., 2024), estimates based on objective measures (i.e. accelerometry) indicate far higher rates of insufficient activity (at times >90%; Tucker et al., 2011). It is therefore unsurprising that physical inactivity remains a leading risk factor for premature death and non‐communicable disease (Katzmarzyk et al., 2021) and exerts a global healthcare burden exceeding US$53 billion per year (Ding et al., 2016). Identifying effective ways to improve physical activity behaviour is therefore a key priority for research.

## NORMS AND THEIR IMPACT ON BEHAVIOUR

Social norms is a broad term that encapsulates distinct subtypes. A key distinction is between descriptive and injunctive norms. Descriptive norms refer to one's perception of the common or typical behaviour of others, whereas injunctive norms refer to one's perceptions of what they ought to do (i.e. the social acceptability of a behaviour; Cialdini et al., 1990). Another distinction, which pertains to the way norms are presented, is between static and dynamic norm messages. As noted above, static norm messages highlight current normative behaviours (e.g. ‘X% of people engage in regular physical activity’ or ‘X is the current average score on this exercise task’). Dynamic norm messages, on the other hand, provide information about a collective change in behaviour over time (e.g. ‘X% of people have increased their physical activity’ or ‘people have tended to improve their score on this exercise task by X% when attempting it a second time’; Sparkman & Walton, 2017, 2019).

Research examining the comparative effects of static and dynamic norm messages has often found that dynamic messages are more effective. For example, Sparkman and Walton (2017) found that participants who received a dynamic norm message stating that the proportion of people who consume meat had *reduced* while queuing at a café were more likely to order a meatless lunch than those who received a static norm message about meat eating, or a control message related to social media use. Sparkman and Walton (2017) also found that dynamic messages were more effective than static messages for promoting environmentally friendly laundry behaviours, while other researchers have found evidence that dynamic messages can reduce people's use of disposable to‐go cups (Loschelder et al., 2019) and encourage them to conserve water (Mortensen et al., 2019).

At the same time, findings in other behavioural domains have been more mixed. For example, Ma and Reynolds‐Tylus (2024) found that attitudes towards not vaping did not differ significantly between participants exposed to static and dynamic norm messages about vaping, but participants in the dynamic norm condition did report greater self‐efficacy to not vape. Graupensperger et al. (2021) found that participants exposed to a dynamic norm message intended to drink fewer drinks per week and engage in heavy episodic drinking less frequently than participants who received a static norm message or no message. However, no significant differences in self‐reported drinking behaviour were observed across conditions 1 month later (see also Geber et al., 2022; Reynolds‐Tylus et al., 2024; Sparkman & Walton, 2019).

## NORMS AND PHYSICAL ACTIVITY BEHAVIOURS

Early norms research in physical activity contexts was primarily correlational and found consistent evidence for a positive relationship between people's perceptions of static descriptive physical activity norms and their own self‐reported physical activity participation (e.g. Ball et al., 2010; Priebe & Spink, 2011). More recently, researchers have used experimental designs to test the causal effects of descriptive norm messages on two distinct sets of physical activity outcomes.

First, researchers have examined the effects on people's physical activity during *daily life*. Here, findings have been mixed. For example, Priebe and Spink (2015) found that office workers reported reducing their sitting time and increasing their stair use and amount they walked around the office after receiving static descriptive norm messages indicating other workers were more active at work than they were. However, Wally and Cameron (2017) found that university students who received static descriptive norm messages (information about the average number of steps other students had taken) did not demonstrate significantly greater subsequent step counts than participants who received no norm messages.

These latter findings perhaps speak to the importance of static norm messages communicating a high norm. Wally and Cameron (2017) provided participants with accurate feedback, which meant that some were informed that others were taking fewer steps than them. Similarly limited effects have been found for messages indicating that a minority of people are engaging in physical activity (e.g. see Crozier & Spink, 2017). Indeed, in the only study that has examined the effect of both static and dynamic norm messages on daily physical activity, Anderson et al. (2024) found that static messages stating that 30% of fellow students are physically active did not improve their student sample's own physical activity behaviours. Interestingly, dynamic and similar ‘trending’ norm messages also showed limited benefits. The trending norm message—which incorporated the features of a dynamic message and additionally specified what the norm was and had changed to (i.e. the proportion of people engaging in physical activity is up from 20% to 30%)—successfully encouraged participants to intermittently stand during long periods of sitting. However, neither it nor the dynamic message reduced participants' engagement in three other sedentary behaviours or improved their overall physical activity relative to static messages or a control.

A second group of studies have examined the effects of norm messages and feedback on people's behaviours during *exercise tasks*. These studies have all communicated high norms and found more consistent findings. For example, Stevens et al. (2023) recruited 102 young adults to complete a simulated home workout task on two occasions. Prior to their second attempt, participants in the static descriptive norm condition were (falsely) told that the number of exercise repetitions they completed during their first attempt was 10% below average, whereas those in the control condition received no feedback. Results showed that participants in the norm condition demonstrated significantly greater increases in the number of exercise repetitions they completed and the maximum heart rate they reached during Trial 2 compared with those in the control condition. Similar effects have been observed in studies using other exercise tasks, including the multistage fitness test (Stevens et al., 2022), wall sit (Guillermo‐Tregoning & Stevens, 2025) and plank (Priebe & Spink, 2014). Notably, although Priebe and Spink (2014) conducted their study before researchers had differentiated between static and dynamic messages, these researchers' manipulation was the only manipulation to demonstrate the hallmarks of a dynamic norm message. Specifically, they informed participants that 80% of similar others had held their second plank for at least 20% *longer* than their first (i.e. implying a tendency for collective change in behaviour). The subsequent improvement in participants' plank performance therefore provides some initial evidence that dynamic norm messages might be an effective way to improve physical activity behaviours during exercise tasks. However, the study provides no insight into how the effects of static and dynamic messages might compare in this context.

## THE PRESENT RESEARCH

We sought to causally test the relative effects of static and dynamic norm messages on a range of physical activity outcomes. Our overarching hypothesis was that participants in the static and dynamic norm conditions in our experiments would demonstrate more positive physical activity outcomes compared with those in the control conditions. As outlined above, although little research has compared the two types of norm messages in physical activity contexts, the trend in research focusing on other behaviours has been for dynamic norm messages to impact behaviour more strongly than static norm messages (e.g. Sparkman & Walton, 2017). We recognised that findings from other behavioural domains (e.g. meat eating and alcohol consumption) may not transfer to physical activity because it differs from many other behaviours in several ways (e.g. in terms of being a behaviour that requires physical effort, is ongoing rather than a ‘one‐off’ and is desirable to increase, not reduce). Nevertheless, we also predicted that the greatest improvements would be demonstrated by participants in the dynamic norm conditions. We also tested potential pathways through which the effects of norm messages on physical activity outcomes might occur.

## STUDY 1

Study 1 was a laboratory experiment. Like recent physical activity studies that have examined the benefits of norm manipulations through laboratory experiments, our physical activity outcomes were participants' exercise output (i.e. performance) and exertion during an exercise task, noting that the health benefits of physical activity (e.g. for metabolic health) can be enhanced to the extent that people exert themselves more and demonstrate greater exercise outputs while undertaking specific forms of physical activity (e.g. see Ross et al., 2015). To provide a methodologically rigorous test of the hypothesised effects that accounted for participants' fitness levels, we used a mixed design. Participants completed two trials of the exercise task, with no norm feedback provided prior to Trial 1, which served as a baseline. Then, prior to Trial 2, participants received either a static norm message, a dynamic norm message or no message (control condition). Reflecting this design, our specific primary hypotheses were that participants in the two norm conditions would demonstrate significantly greater *improvements* in exercise task performance (H1a) and exertion (H1b) from Trial 1 to Trial 2, with the greatest improvements apparent in the dynamic norm condition.

We also examined two potential mediators of these effects. First, increased *task motivation* has been shown to mediate the effect of static norm messages on exercise performance and exertion (Stevens et al., 2023). Extending on these findings, we hypothesised that participants in the two norm conditions would experience a greater increase in task motivation from Trial 1 to Trial 2 compared with participants in the control condition, and that increases in task motivation would be a positive predictor of greater improvements in performance and exertion, resulting in significant indirect effects (H2).

Second, Priebe and Spink (2014) found that participants' *self‐efficacy* in relation to their exercise task increased after they received a dynamic norm message. Moreover, although self‐efficacy's role as a possible mediator of the effects of norm messages on physical activity outcomes has not been tested, self‐efficacy has been shown to mediate the effects of dynamic norm messages on attitudes and intentions related to other behaviours (e.g. vaping and quitting smoking; Ma & Reynolds‐Tylus, 2024; Sparkman & Walton, 2019). We therefore hypothesised that participants in the two norm conditions would report greater self‐efficacy in relation to the exercise task compared with participants in the control condition, and that higher self‐efficacy would be a positive predictor of greater improvements in performance and exertion, also resulting in significant indirect effects (H3).

## METHOD

### Participants

We recruited 105 students from an Australian university (71 women, 33 men, 1 non‐binary; *M*_age_ = 21.96 years, *SD* = 2.65, range = 18–32; 74% Asian, 21% Caucasian, 5% other). Participants were required to be physically capable of undertaking the exercise task and have no health condition(s) that may have heightened their risk of experiencing an adverse medical event while doing so. These requirements were stated in the recruitment materials and verified through a health screening questionnaire (the Adult Pre‐Exercise Screening System; Exercise & Sport Science Australia, 2024) that participants completed prior to study enrolment. Participants were recruited through the psychology research participation scheme at the host institution and received their choice of either course credit or AUD$10 for participating. We did not assess participants' physical activity experience or participation and noted in the study advertisement that the study was open to people of all fitness levels. Nevertheless, the advertisement likely primarily appealed to people with an interest in exercise, and we would characterise the sample overall as recreationally active.

### Sample size justification

The target sample size of 105 was informed by *a priori* power analyses. Research examining the effect of norm manipulations on exercise behaviours has typically found large effect sizes. In their study comparing the effect of a static descriptive norm manipulation to a control condition on participants' performance during a simulated home workout, Stevens et al. (2023) found a large effect (*η*_*p*_^2^ = .25, equivalent to *f* = 0.57). Similarly, Priebe and Spink (2014) found a large effect (*η*_*p*_^2^ = .22, equivalent to *f* = 0.53) when they compared the effect of a dynamic descriptive norm manipulation to a control condition on participants' performance on a plank exercise task. Using these effect sizes and an *α* of .05, *a priori* power analyses in G*Power (Faul et al., 2007) indicated that sample sizes of just 36 and 42 would be required to achieve 80% power in analyses of the two‐way interaction between time (Trial 1–Trial 2) and condition that were planned to test the primary hypotheses (H1a and H1b). However, noting that effect sizes have at times been smaller in studies that have compared the effects of dynamic and static messages in other contexts (e.g. Sparkman & Walton, 2017), and taking our available practical resources into account (see Lakens, 2022), we opted for a more conservative approach and targeted a sample size of 105 participants. This target also reflected a desire to maximise power in our mediation analyses. There was limited previous research to draw on for a power analysis for this. No research has examined whether task motivation or self‐efficacy mediate the relative effects of static and dynamic norm messages on physical activity behaviours. Indeed, no research has examined whether self‐efficacy mediates the effects of *any* norm messages in physical activity contexts. However, Stevens et al. (2023) tested whether task motivation mediated the effect of static norm feedback on exercise task performance (compared with a control). Using the standardised path coefficients and standard deviations these researchers reported, we conducted a Monte Carlo power analysis with 1000 replications using the MARlab application (Schoemann et al., 2017). Providing further evidence that our sample was sufficiently powered, this indicated that 69 participants would be required to achieve 80% power. Nevertheless, the results from our mediation models should be interpreted with some caution given that they also included self‐efficacy. Our target sample, hypotheses, and planned analyses were pre‐registered prior to data collection—see https://aspredicted.org/9DS_YL3.

### Procedure

Ethical approval for the study was obtained from the Human Research Ethics Committee at the first author's institution. Once participants' eligibility had been confirmed via the health screening questionnaire, they were invited to book a time to participate and randomly assigned to one of the three experimental conditions using a random number generator. At the beginning of their testing session, the experimenter (the third author) summarised what the session would entail. Specifically, participants were informed that they would be asked to complete two 2‐min cycling time trials, separated by a 5‐min break,^1^ and answer some related survey questions. The experimenter explained that the aim was to travel the furthest distance possible during each trial. At this stage, participants provided written consent if they wished to take part. Following this, they were asked to attach a heart rate monitor and complete a task motivation measure (see below). Participants were then given time to adjust the cycling ergometer (a Wattbike Trainer) to their preferences and complete a self‐paced warm‐up. The air brake on the Wattbike was set to level 6 and the magnetic brake to level 2 (equivalent to a flat surface) for all participants to ensure consistency (see also McCluskey & Stevens, 2025). The experimenter explained where information pertaining to the distance they had covered and the time elapsed would be visible on the bike's monitor and repeated that the objective was to travel as far as possible within the 2 min. When participants indicated they were ready, Trial 1 began.

During the 5‐min break after Trial 1, participants were first asked to complete two tasks designed to make their social identity (i.e. sense of self) as a member of the norm reference group (a student at their university) salient, noting evidence that this can enhance the effectiveness of norm manipulations (e.g. Liu et al., 2019).^2^ First, they were presented with a series of statements related to being a student at their university and instructed to tick the statements they agreed with (see Greenaway et al., 2015). Second, they completed Leach et al.’s (2008) 14‐item social identity scale in relation to their social identity as a student at their university. Participants in the two norm conditions were then exposed to the experimental manipulation (see below), and all participants completed the same single‐item task motivation measure as they did prior to Trial 1, as well as a self‐efficacy measure. Before Trial 2 commenced, participants were reminded that the goal was to travel as far as possible in the 2‐min. Trial 2 then began. The experimenter did not communicate with participants during either trial. After Trial 2, all participants completed a demographic questionnaire, and those in the norm conditions also completed a manipulation and suspicion check. Finally, all participants were debriefed (verbally and in writing) and provided with the opportunity to withdraw their data if they wished, which none did.

### Experimental manipulation

**FIGURE 1:**
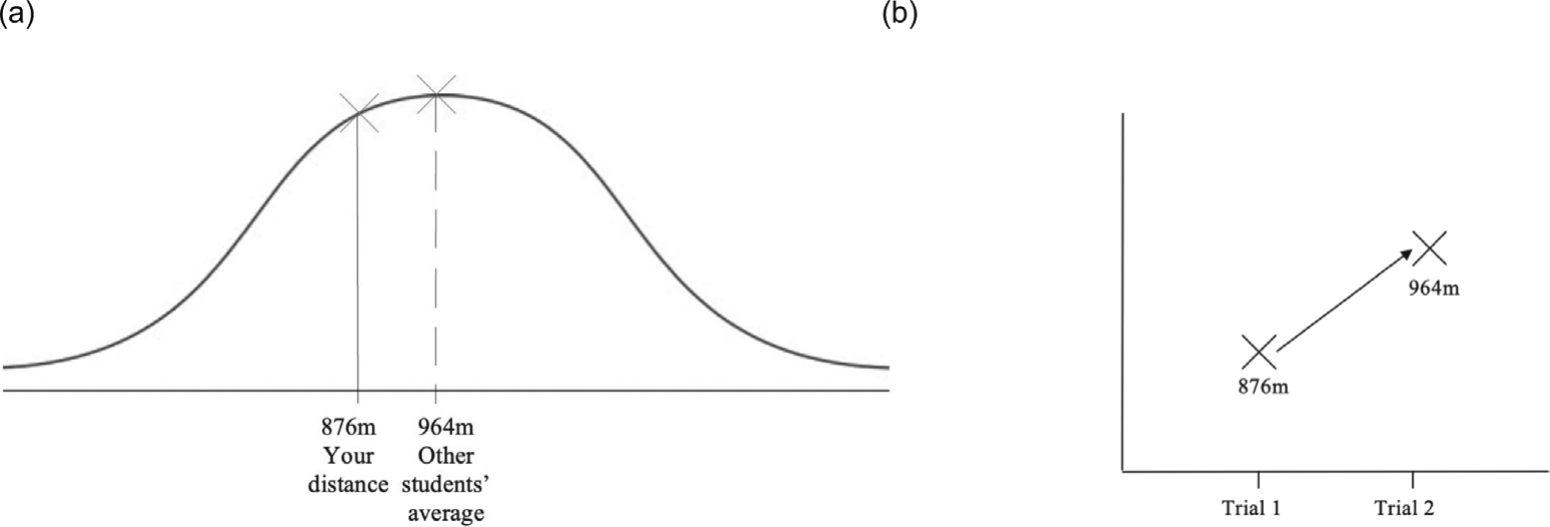
Examples of the figures drawn to support the (a) static and (b) dynamic norm manipulations in Study 1.

For the experimental manipulation, participants in both conditions were first told that they would be provided with an update on the results of the study. Following this, participants in the static norm condition were told that the distance they travelled in Trial 1 was approximately 10% below the average distance that other students at their university who had completed the study so far had travelled in their first trial. Mirroring previous research (Guillermo‐Tregoning & Stevens, 2025; Stevens et al., 2022, 2023), the experimenter also drew this information on a bell curve as they provided it. That is, the experimenter marked a cross on the bell curve to denote the ‘average’ distance travelled by other students and another to the left of this to denote the distance that the participant travelled in Trial 1. No norms were calculated, but the feedback was personalised such that the ‘average’ distance was always calculated by adding 10% to each participant's Trial 1 distance. The experimenter also wrote these numerical distances next to each of the crosses on the bell curve (see Figure 1a for an example).

Participants in the dynamic norm condition were told that the main finding of the study so far was that other students had tended to travel 10% further during their second trial compared with their first. Again, this feedback was reinforced visually. The experimenter first drew a cross and next to this marked the distance the participant had travelled in Trial 1. They then drew an upward arrow from this to a second cross. Alongside this, they wrote the distance that the participant would travel if they followed ‘the typical pattern of other students’. This distance was calculated by computing 10% of each participant's Trial 1 distance and adding this to their Trial 1 distance (see Figure 1b for an example). This manipulation was informed by previous dynamic norm manipulations in other contexts (e.g. Ma & Reynolds‐Tylus, 2024) but developed specifically to suit the exercise task context, while retaining the crucial characteristic of implying collective *change in behaviour* (specifically across the two trials, reflected by a tendency for people to improve their performance).

Mirroring the approach used in other norm research (e.g. Stevens et al., 2022; Wally & Cameron, 2017), both manipulations therefore included a feedback element in addition to the key characteristic of a static or dynamic norm message to help tie the messages to participants' own performance. That is, in the static norm condition, we additionally stated how the norm compared with participants' own Trial 1 score, and in the dynamic norm condition, we additionally stated the score the participant would achieve if they followed the pattern of others. We refer to the norm conditions as the ‘static norm message’ and ‘dynamic norm message’ conditions for brevity but note that they could be more accurately considered ‘static/dynamic norm messages + feedback’ conditions (and return to the point in the General Discussion). A 10% differential was chosen for both manipulations because it was considered a realistic improvement for participants to achieve on their second trial, noting previous evidence of strong norm convergence using this differential (Stevens et al., 2023).

### Measures

#### Task motivation

Consistent with previous research seeking to assess the magnitude of participants' motivation for a specific exercise task (e.g. Hutchinson et al., 2011; Stevens et al., 2023), we assessed participants' motivation for each trial using a single‐item scale: ‘How motivated are you to exert maximum effort during the cycling task you are about to complete?’ Participants responded to the item on an 11‐point scale ranging from 0 (*not at all motivated*) to 10 (*extremely motivated*).

#### Self‐efficacy

Self‐efficacy was assessed using a 5‐item scale adapted from Priebe and Spink (2014), who assessed self‐efficacy in relation to a plank task. Consistent with these researchers' approach, the 5 items asked participants to indicate their confidence that, in Trial 2, they would perform close to, as well as, or better than they did in Trial 1 on 11‐point scales ranging from 0 (*not at all confident*) to 10 (*extremely confident*). Specifically, Items 1 and 2 asked participants how confident they were they would be able to travel within 20% and 10% respectively of the distance they travelled in Trial 1, Item 3 asked participants how confident they were that they would be able to travel the same distance as in Trial 1, and Items 4 and 5 asked participants how confident they were they would be able to travel at least 10% and 20% further respectively than in Trial 1. Consistent with Priebe and Spink's (2014) approach, responses to the five items were averaged, such that a higher score indicated greater self‐efficacy. The measure demonstrated good internal reliability (*α* = .82).

#### Exercise performance

We recorded the distance participants travelled during the time trials (in metres) as an objective measure of performance. The Wattbike was programmed to start recording distance when the researcher pressed ‘start’ at the beginning of each 2‐min trial and to stop recording automatically once 2 min elapsed. The total distance was obtained from the data file generated by the Wattbike.

#### Exertion

Two objective measures of exertion were obtained: participants' *maximum heart rate* and *average heart rate* during the trials (both measured in beats per minute). To facilitate this, participants wore a POLAR H10 heart rate monitor throughout the testing session. This was linked to the Wattbike via Bluetooth, enabling participants' heart rate to be recorded continuously throughout both trials. The maximum and average heart rate of each participant were extracted from the resulting data files generated by the Wattbike.

#### Manipulation and suspicion checks

Reflecting the different information they were given, we developed separate manipulation checks for participants in the two norm conditions. Participants in the static norm condition were asked to indicate how they were told their cycling performance in Trial 1 compared with the average for fellow students at their university. The response options were as follows: ‘I was below average’ (the correct response), ‘I was above average’, ‘I was at the average’, ‘I was not told’ and ‘I cannot remember’. Participants in the dynamic norm condition were asked to indicate how they were told fellow students at their university usually perform in Trial 2 compared with Trial 1. Response options were as follows: ‘People tend to perform better’ (the correct response), ‘People tend to perform worse’, ‘People tend to perform the same’, ‘I was not told’ and ‘I cannot remember’. All participants were also given an open‐ended suspicion check that asked them to indicate what they thought the main purpose of the experiment was.

## RESULTS

### Preliminary analyses

All analyses were conducted in IBM SPSS Statistics (version 30). No participants correctly identified the purpose of the study, and thus, all passed the suspicion check. Two participants in the dynamic norm condition failed the manipulation check and were excluded from all analyses. There were no missing or implausible (i.e. out of range) values. Data were also screened for outliers and indices of nonnormality. Consistent with the approach we used in our main analyses (see below), outliers were defined as participants who showed extreme or unusual changes from Trial 1 to Trial 2, rather than atypical values in either individual trial (apart from for the self‐efficacy variable, which was only assessed once). Four scores (each on different variables) exceeded the *Z* > ±3.29 criteria for univariate outliers (Tabachnick & Fidell, 2019). All scores were within range and reflected plausible responses to the experimental protocol (e.g. a participant who demonstrated a particularly large improvement from Trial 1 to Trial 2). They were therefore retained in the analyses. All variables were approximately normally distributed, with all skewness and kurtosis values within the ±2 and ±7 cut‐offs (Byrne, 2016).

To examine whether baseline differences existed between participants in the three conditions, we conducted one‐way analyses of variance (ANOVAs) on the Trial 1 scores. There were no significant differences between the conditions for Trial 1 distance (*F*(2, 100) = 1.46, *p* = .237, *η*_*p*_^2^ = .028), maximum heart rate (*F*(2, 100) = .60, *p* = .551, *η*_*p*_^2^ = .012), average heart rate (*F*(2, 100) = 1.29, *p* = .280, *η*_*p*_^2^ = .025) or task motivation (*F*(2, 100) = .722, *p* = .488, *η*_*p*_^2^ = .014). One‐way ANOVAs also indicated that, across conditions, there were no significant differences in participants' social identification with the norm reference group (*F*(2, 100) = .07, *p* = .929, *η*_*p*_^2^ = .001) or age (*F*(2, 100) = 2.27, *p* = .109, *η*_*p*_^2^ = .043). A Chi‐square test of independence (excluding the participant who identified as non‐binary, because their inclusion violated the assumptions of the test) indicated that gender distribution did not differ significantly across the experimental conditions, *χ* ^2^(2, *N* = 102) = 0.12, *p* = .94. Means and standard deviations for all variables across experimental conditions and trials are shown in Table 1.

**TABLE 1:** Descriptive statistics for the study variables across experimental conditions and trials in Study 1.

Variable	Control condition				Static norm condition				Dynamic norm condition			
	Trial 1		Trial 2		Trial 1		Trial 2		Trial 1		Trial 2	
	*M*	*SD*	*M*	*SD*	*M*	*SD*	*M*	*SD*	*M*	*SD*	*M*	*SD*
Performance	1077.41	164.05	1056.86	160.58	1053.33	195.94	1097.20	160.07	1123.24	147.42	1130.10	124.73
Maximum heart rate	167.03	19.80	173.51	16.34	171.34	22.15	181.54	15.21	171.36	13.73	180.21	10.02
Average heart rate	143.69	18.56	154.14	17.19	150.26	20.85	162.80	17.94	149.45	15.94	162.30	12.44
Task motivation	7.86	1.46	7.57	1.31	7.39	1.87	8.23	1.73	7.50	1.78	8.32	1.44
Self‐efficacy	—	—	5.49	2.11	—	—	6.20	2.11	—	—	5.94	1.29
Social identification	—	—	5.08	0.92	—	—	5.15	0.84	—	—	5.09	0.69

### Main analyses

#### Hypotheses 1a and 1b: The effect of norm condition on performance and exertion

To test H1a and H1b, we conducted three mixed model ANOVAs (i.e. one for each outcome). Time (Trial 1–Trial 2) was the within‐subjects factor and experimental condition (control–dynamic norm–static norm) was the between‐subjects independent variable. These analyses test how groups differ, on average, in changes from Trial 1 to Trial 2 (the focal question in this study) and were thus preferred to ANCOVAs with Trial 1 scores entered as covariates, which focus on how participants differ at Trial 2, adjusting for their Trial 1 scores (see Smolkowski, 2019). Consistent with this approach, we followed up significant interactions with one‐way ANOVAs that compared the change scores for the dependent variables from Trial 1 to Trial 2 (i.e. after subtracting participants' Trial 1 scores from their Trial 2 scores). These analyses are conceptually and numerically equivalent to mixed ANOVAs (see Anderson et al., 1980) but enable specific comparisons between individual pairs of experimental conditions. Consistent with recommendations (Rubin, 2021), the Bonferroni correction was applied to analyses that fell under the same joint hypothesis, meaning that for the exertion outcomes alpha was set at .025.

In the performance model, the main effect for time was not significant, *F*(1, 100) = 1.15, *p* = .287, *η*_*p*_^2^ = .01. However, there was a significant time × condition interaction, *F*(2, 100) = 4.02, *p* = .021, *η*_*p*_^2^ = .07. Planned post hoc pairwise comparisons with Tukey's (1949) correction revealed that participants in the static condition improved the distance they travelled to a greater extent than those in the control condition, *t*(68) = 2.83, *p* = .016. There was no significant difference in terms of improvements between those in the dynamic and control conditions (*t*(66) = 1.19, *p* = .465) or those in the static and dynamic conditions (*t*(66) = 1.60, *p* = .251). Simple slope analyses demonstrated that the change in performance over time was significant in the static condition (*p* = .008) but not in the dynamic condition (*p* = .680) nor the control condition (*p* = .205).

In both exertion models, the main effect for time was significant (maximum heart rate: *F*(1, 100) = 48.35, *p* < .001, *η*_*p*_^2^ = .33; average heart rate: *F*(1, 100) = 94.62, *p* < .001, *η*_*p*_^2^ = .49) such that, across conditions, participants tended to demonstrate higher exertion in Trial 2 than Trial 1. However, contrary to H1b, the time × condition interaction was not significant in either model (maximum heart rate: *F*(2, 100) = .80, *p* = .452, *η*_*p*_^2^ = .02; average heart rate: *F*(2, 100) = .38, *p* = .687, *η*_*p*_^2^ = .01), indicating that changes in exertion from Trial 1 to Trial 2 did not differ significantly between participants in the three conditions. Therefore, no follow‐up tests were conducted.

#### Hypotheses 2 and 3: The mediating role of task motivation and self‐efficacy

To assess H2 and H3, we used PROCESS (Model 4; Hayes, 2017) to test three parallel mediation models (i.e. one for each dependent variable). These assessed the indirect effects of experimental condition on performance and the two exertion indicators through participants' task motivation (H2) and self‐efficacy (H3). We used bias‐corrected bootstrapping with 5000 resamples to calculate 95% confidence intervals for the indirect effects, with a significant mediation effect indicated if the confidence interval did not cross zero (Hayes, 2017). Given that the predictor variable was multicategorical (i.e. comprising three conditions), we used the parallel mediation method for multicategorical independent variables detailed by Hayes and Preacher (2014). This requires a reference condition to be selected. We chose the control condition because this enabled us to include in our models the comparisons between this and both the norm conditions, noting that we expected both types of norm message to enhance participants' motivation and self‐efficacy relative to no message. To achieve this, we coded control = 0 in our indicator coding scheme. This meant that PROCESS coded the control condition as 0 in both vectors it created (Vector 1: control = 0, static = 1, dynamic = 0; Vector 2: control = 0, static = 0, dynamic = 1) and thus that the mediation models estimated the *relative* indirect effects of both the static and dynamic conditions compared with the control condition on the outcomes via the mediators. More specifically, consistent with our approach to testing H1, and our primary interest in assessing whether the static and/or dynamic norm messages changed participants' performance and exertion, we used change scores for both the dependent variables and task motivation (which we also assessed twice). Again, we calculated these by subtracting participants' Trial 1 scores from their Trial 2 scores. The mediation models therefore tested whether the experimental condition participants were in predicted a change in their performance and exertion through (a) their self‐efficacy and/or (b) a change in their task motivation.

Full results from the two mediation models are shown in Table 2. Consistent with H2, there were significant indirect effects through task motivation. Specifically, being in both the static and dynamic conditions compared with the control predicted greater improvements in performance and maximum, but not average, heart rate via increases in task motivation. Regarding H3, all effects related to the self‐efficacy mediation pathways were in the hypothesised direction and self‐efficacy significantly predicted improvements in all three outcomes. However, no condition comparisons significantly predicted self‐efficacy, and all indirect effects were non‐significant, meaning H3 was not supported.^3^

**TABLE 2:** Full results of the mediation models testing Hypotheses 2 and 3 in Study 1.

Outcome	Comparison	Mediators										Direct effects		Total effects	
		Task motivation (change)					Self‐efficacy								
		Condition → mediator		Mediator → outcome		Indirect effect *β* [95% CI]	Condition → mediator		Mediator → outcome		Indirect effect *β* [95% CI				
		*β*	*p*	*β*	*p*		*β*	*p*	*β*	*p*		*β*	*p*	*β*	*p*
Performance	Control vs. static	.80	<.001	.26	.002	.21 [.06, .38]	.38	.12	.50	<.001	.19 [−.08, .46]	.26	.18	.66	.006
	Control vs. dynamic	.78	<.001	.26	.002	.21 [.06, .39]	.24	.33	.50	<.001	.12 [−.10, .34]	−.04	.82	.28	.24
Maximum heart rate	Control vs. static	.80	<.001	.23	.026	.18 [.02, .37]	.38	.12	.32	.001	.12 [−.05, .39]	−.004	.99	.30	.21
	Control vs. dynamic	.78	<.001	.23	.026	.18 [.01, .38]	.24	.33	.32	.001	.08 [−.09, .28]	−.06	.78	.19	.43
Average heart rate	Control vs. static	.80	<.001	.16	.15	.12 [−.05, .31]	.38	.12	.27	.01	.10 [−.05, .32]	−.06	.82	.17	.49
	Control vs. dynamic	.78	<.001	.16	.15	.12 [−.06, .31]	.24	.33	.27	.01	.06 [−.06, .22]	.009	.97	.19	.43

## DISCUSSION

In Study 1, we tested the relative effects of static and dynamic norm messages on physical activity outcomes for the first time. Our hypotheses were partially supported. Participants who received static norm messages demonstrated greater improvements in exercise performance than those in the control condition, who received no norm messages. However, participants who received dynamic norm messages did not improve their performance to a significantly greater extent than those in the control condition and tended to demonstrate smaller improvements than those in the static condition, with this difference also non‐significant. H1b was not supported: There were no significant differences between conditions for changes in exertion. However, supporting H2, both types of norm message had a positive *indirect* effect on both performance and one of the two exertion indicators through their positive impact on participants' task motivation. H3 was not supported, with all indirect effects through self‐efficacy non‐significant. The trends observed were, however, consistent with the hypothesised effects: Both types of norm messages were non‐significant but positive predictors of self‐efficacy, which, in turn, was a significant positive predictor of improvements in performance and exertion.

Building on previous research (e.g. Priebe & Spink, 2014; Stevens et al., 2023), findings therefore provide further evidence that both types of norm messages can positively impact people's physical activity behaviours. However, contrary to the typical trend in research that has focused on other behaviours (e.g. Sparkman & Walton, 2017), static norm messages had a stronger effect, with the effects of the dynamic messages occurring only via their positive effect on participants' task motivation—indirect effects that were also observed for static norm messages. The direct and indirect effects of both types of messages also tended to be largest for the performance outcome, with those related to maximum and average heart rate smaller and more commonly non‐significant. This could be partly due to the study protocol (i.e. two trials within the same experimental session) and nature of the exertion indicators. Participants across all conditions tended to demonstrate a higher maximum and average heart rate during Trial 2 than Trial 1, most likely because their heart rate was already elevated, and they were already experiencing some fatigue, before they began Trial 2. This could have played a role in limiting the magnitude of the differences between the conditions on the exertion indicators.

Study 1 had several strengths, including the mixed experimental design and objective measures of the outcome variables. However, it also had some limitations. First, the sample comprised university students who were primarily female, of a similar age, and likely both relatively homogenous in terms of other sociodemographic characteristics and their interest in physical activity (having selected to participate in a study involving an exercise task). Along these lines, it is also possible that our sample may have differed from the broader population in other ways that might have magnified or suppressed the effect of the manipulations (e.g. in terms of their competitiveness). Generalising the results to adults more broadly should therefore be done with caution. Second, although the laboratory setting facilitated a highly controlled test of the causal effects of the two types of norm messages, it limited the ecological validity of the findings. To help address this and gain a broader understanding of the effect of the two types of messages on physical activity behaviours, in Study 2, we focused on their potential impact on people's physical activity during daily life.

## STUDY 2

More specifically, in Study 2, we focused on how the two types of norm messages might impact *older adults'* physical activity. In particular, we focused on adults aged 65 and over, in line with the age at which people are often classified as older adults according to physical activity guidelines (e.g. see National Health Service, 2024). The choice to focus on older adults was driven by a recognition that (a) no research has sought to examine whether norm messages (of either type) could be harnessed to improve physical activity behaviours in this population, and (b) physical inactivity is a particular concern among older adults: The most recent global data indicate that rates of insufficient activity are 12.2% higher among people aged 60+ compared with those aged 18–60 (Strain et al., 2024).

We again used a mixed experimental design. At Time 1, participants reported their physical activity. They were then randomly assigned to either the dynamic norm, static norm or control condition. We then obtained two measures of the proximal impact of the norm manipulations: (a) on their physical activity intentions and (b) on their interest in becoming more physically active. One week later, we assessed participants' physical activity behaviours again to facilitate an analysis of whether the norm manipulations had changed their behaviour from Time 1. Our primary hypotheses were that participants in the two norm conditions would demonstrate significantly greater physical activity intentions (H1a), interest in becoming more active (H1b) and improvements in their physical activity behaviours (H1c) than participants in the control condition, with the strongest effects apparent in the dynamic norm condition (although the results from Study 1 made us less confident of this).

We again also examined two potential mediators. First, we again explored the role of self‐efficacy, noting that although the indirect effects through self‐efficacy were non‐significant in Study 1, they were in the hypothesised direction and self‐efficacy was a significant predictor of each of the physical activity outcomes. We therefore hypothesised significant indirect effects through self‐efficacy (this time in relation to engaging in exercise in general), such that participants in the two norm conditions would report higher levels of self‐efficacy and self‐efficacy would be a positive predictor of the three physical activity outcomes (H2).

Task motivation was not relevant for Study 2 because there was no focal task involved in the study. We therefore examined a new potential mediator: fear of pain while exercising. This is a key barrier to physical activity participation among older adults in particular and thus a strong predictor of their physical activity participation (e.g. see Larsson et al., 2016). The potential for norm messages to help alleviate people's fear of pain while exercising has not been examined, and this hypothesis was therefore more exploratory. However, we reasoned that incorporating norm messages that spoke to how it is (increasingly) common for people to use physical activity for *pain management* might help achieve the goal of alleviating their fear of pain while exercising (noting growing evidence that exercise can help treat pain; e.g. Lima et al., 2017). Our final hypothesis was therefore that there would be significant indirect effects through fear of pain while exercising, such that participants in the two norm conditions would report lower fear of pain and fear of pain would be a negative predictor of the three physical activity outcomes (H3).

## METHOD

### Participants

We recruited 163 older adults via the online research platform Prolific (89 men, 73 women, 1 preferred not to say; *M*_age_ = 70.18 years, *SD* = 4.64, range = 65–82; 98% British). Consistent with standard Prolific rates, participants received a total of £1.80 for participating—£1.50 for the first survey and £0.30 for the (much shorter) second survey.

### Sample size justification

We aimed to recruit a slightly larger sample than in Study 1, again considering our resources (Lakens, 2022), as well as the results from Study 1. Along these lines, using the effect size we observed for the effect of the norm messages on performance in Study 1 (*η*_*p*_^2^ = .07, equivalent to an *f* = .27), an *a priori* power analysis indicated that a sample size of 141 would be required to achieve 80% power in an analysis of the time × condition interaction that was planned to test the effect of the messages on the key outcome: change in physical activity behaviour. Notably too, power analyses using the effect sizes reported by Priebe and Spink (2015) in their tests of the effects of static norm messages on three *everyday* physical activity behaviours among office workers (their sitting time, use of the stairs rather than the lift and walking around the office) suggested that 33, 129 and 156 participants would be required respectively. Similarly, using the effect size reported by Anderson et al. (2024) in their test of the relative effects of static, dynamic, trending and no norm messages on encouraging people to stand during extended periods of sitting indicated that 135 participants would be required. Our resources enabled us to target a sample size of 150 and to incorporate contingencies to help us reach this target at both timepoints. Specifically, to account for potential attrition across the two timepoints, and after noting that one participant had failed the manipulation check during a preliminary check of the data after activating the study, we recruited an additional 13 participants at Time 1.

Again, there was limited previous research from which to draw for a power analysis for the planned mediation analyses. No research has examined fear of pain while exercising as a possible mediator of the effects of norm messages on physical activity outcomes. However, drawing on the standardised path coefficients and standard deviations related to the self‐efficacy pathway in Study 1 (specifically those for the dynamic vs. control group comparison to be conservative because these effects were smallest), a Monte Carlo power analysis with 1000 replications in the MARlab application (Schoemann et al., 2017) indicated 135 participants would be required to achieve 80% power. Similar to Study 1, although this aligns with our sample size target, the results from the mediation models should be interpreted with some caution given that these models also included fear of pain while exercising. We again pre‐registered our target sample size, hypotheses and planned analyses prior to data collection—see https://aspredicted.org/4cnd-v5jc.pdf.

### Procedure

Ethical approval for the study was obtained from the Human Research Ethics Committee at the first author's institution. We advertised the study on Prolific as a two‐part, online study examining the healthy lifestyles of older adults. The advertisement stated that participants would be asked to complete two online surveys, 1 week apart, containing questions about their physical activity habits and experiences. We used Prolific's eligibility screening function to make the study available only to Prolific users aged 65 and over.

Participants began Survey 1 by indicating their age, gender, nationality and Prolific ID (which enabled us to match their two survey responses). They then completed the physical activity measure for the first time (which pertained to their activity over the past week). Like in Study 1, we sought to make participants' social identity as a member of the norm reference group (older adults) salient prior to the experimental manipulation and therefore next asked participants to complete Leach et al.'s (2008) social identity scale. We then presented participants with norm messages relevant to the condition they had been randomly assigned to (or no messages if they were in the control condition). Randomisation was achieved by embedding a random number generator within the survey. Participants then completed measures of self‐efficacy, fear of pain while exercising and physical activity intentions. Participants in the dynamic and static norm conditions then completed a manipulation check. Finally, all participants were presented with an information page about physical activity and ways to become more active. The time participants spent on this page served as our measure of participants' interest in becoming more active. Further details related to this measure, the other measures and the norm messages are provided below.

We made Survey 2 available for 48 h 1 week after Survey 1 and sent all participants a message through Prolific informing them that it was open. Survey 2 contained just one measure—the same physical activity measure used in Survey 1, again framed to capture activity over the past week. Following this, a debrief page outlining the study aims and methods was displayed. Finally, participants were provided with an opportunity to withdraw their data, which no participant chose to do.

### Experimental manipulation

In developing the norm messages, we again drew on previous research (e.g. Ma & Reynolds‐Tylus, 2024) to guide the phrasing and presentation but constructed them to specifically reflect their intended goals. Specifically, we developed three messages of each type, which aimed to target participants' normative perceptions of older adults' physical activity participation, confidence (i.e. self‐efficacy) in their ability to be active and use of exercise for pain *relief* (i.e. aimed at helping reduce their fear of pain while exercising). The messages were disguised as information about the latest research on the healthy lifestyles of older adults and presented on consecutive pages. The messages were framed differently according to condition, such that they either communicated the current norm (static condition) or a collective change in behaviour over time (dynamic condition). However, like in Study 1, we used identical percentages. For example, the static norm message related to physical activity participation indicated that a study had found that 79% of older adults over 65 are exercising regularly, whereas the equivalent dynamic norm message indicated that a study had found that 79% of older adults aged over 65 were exercising more regularly than they did when they were 55. The self‐efficacy and exercise for pain relief messages similarly used fictional high percentages. Bold font and graphs were used to highlight key information and provide a visual representation of what the messages indicated. Images were also included to make the content more visually appealing. Full copies of the pages of the survey that contained the manipulations are provided in Data S1. We also note that the information participants received as part of the physical activity interest measure regarding the benefits of physical activity and how it can be incorporated into daily life may have interacted with the manipulation to impact physical activity behaviour (e.g. by helping participants understand how to enact a desire created by the messages to increase their physical activity). This should therefore be considered when appraising the results related to this outcome and is discussed further in General Discussion.

### Measures

#### Physical activity behaviour

Participants' physical activity in the last week was measured in both surveys using the Godin–Shephard Leisure‐Time Physical Activity Questionnaire (GSLTPAQ; Godin & Shephard, 1985). Participants were asked to report the number of times in the last week they had engaged in strenuous, moderate and mild physical activity for more than 15 min at a time. To aid clarity, we provided examples of the different types of activity, as well as an example of a fictitious person's physical activity throughout a week and how that person would complete the measure. Scoring the GSLTPAQ involves multiplying the frequencies of strenuous, moderate and mild activities by standard metabolic equivalent energy expenditure values (nine, five and three, respectively) and summing the resulting values to compute a total score—the leisure score index. This measure, like all self‐report physical activity measures, has limitations. Perhaps most notably, contrary to many country's guidelines, which focus on total minutes of physical activity per week, it only counts exercise bouts lasting >15 min (and does not differentiate between bouts lasting just over 15 min and those that last much longer). However, the leisure score index has been validated against objective physical fitness indicators (e.g. VO_2_ max; Godin & Shephard, 1985) and objectively estimated energy expenditure values (Miller et al., 1994).

#### Physical activity intentions

To measure participants' physical activity intentions, we adapted the GSLTPAQ (Godin & Shephard, 1985) such that participants were asked to report the number of times they *intended* to engage in strenuous, moderate and mild physical activity for more than 15 min at a time in the *next week*. Mirroring the approach used for the physical activity behaviour measure, a total intentions score was calculated by multiplying the strenuous, moderate and mild scores by nine, five and three respectively and summing the totals.

#### Physical activity interest

At the end of Survey 1, participants were presented with a page that included information about the benefits of being physically active, physical activity guidelines for older adults and what they mean in practice and age‐appropriate advice on how to incorporate physical activity into everyday life (e.g. using stairs instead of lifts or escalators). The page included 694 words and had seven videos embedded within it related to these topics (e.g. a 10‐min guided living room workout from the British Heart Foundation; see Data S2). Using an embedded timer, we recorded the time participants spent on this page in seconds as a behavioural indicator of their interest in becoming more active (such that greater time indicated greater interest). We estimated that the maximum time a participant could spend actively engaging with this content (watching all videos fully and reading all the words at 238 words per minute; Brysbaert, 2019) was 1849 s (approximately 31 min).

#### Self‐efficacy

We measured self‐efficacy using Kroll et al.'s (2007) 10‐item Exercise Self‐Efficacy Scale (e.g. ‘I am confident that I can find means and ways to be physically active and exercise’). Participants recorded their responses on a 4‐point scale ranging from 1 (*never true*) to 4 (*always true*). A mean score was calculated, with higher scores indicating greater self‐efficacy. Although this measure differs from some other self‐efficacy measures (which ask participants to rate their confidence on wider response scales, e.g. Marcus et al., 1992), it has demonstrated strong content and construct validity and internal consistency (Kroll et al., 2007). Internal consistency was also excellent in our sample (*α* = .95).

#### Fear of pain while exercising

We measured fear of pain while exercising using an adapted 14‐item version of the 17‐item Tampa Scale for Kinesiophobia (Miller et al., 1991). The original Tampa Scale was primarily designed to capture fear of pain while exercising among people with existing health conditions, and thus, several of the items assume the respondent has pain (e.g. ‘My pain would probably be relieved if I were to exercise’). Given that we recruited a general sample of older adults, many of whom would not have had a condition that caused them pain, we either adapted these items to remove this assumption (e.g. the example item above was adapted to ‘If I were in pain, it would probably be relieved if I were to exercise’) or removed them in instances where this was not possible (e.g. ‘My accident has put my body at risk for the rest of my life’ was removed). Participants recorded their responses on 4‐point scales ranging from 1 (*strongly disagree*) to 4 (*strongly agree*). A mean score was calculated, with a higher score indicating greater fear of pain while exercising. The measure demonstrated good internal consistency (*α* = .85).

#### Manipulation check

For the manipulation check, participants in the static and dynamic norm conditions were prompted to answer a question about older adults. In the static norm condition, participants were asked ‘Based on the information you were given in this survey, most older adults are … (a) active, confident about doing exercise, and finding exercise helps with their pain (the correct answer), (b) inactive, lacking confidence about doing exercise, and struggling with their pain while exercising, or (c) I can't remember’. In the dynamic condition, participants were asked ‘Based on the information you were given in this survey, most older adults are … (a) getting more active, more confident about being active, and finding that their pain is improving while exercising (the correct answer), (b) getting less active, less confident about being active, and finding that their pain is getting worse during exercise, or (c) I can't remember’.

## RESULTS

### Preliminary analyses

All analyses were again conducted in IBM SPSS Statistics (version 30). One participant (in the static condition) failed the manipulation check and was excluded from all analyses. Of the 163 participants who completed Survey 1, 150 completed Survey 2. Analyses below including physical activity behaviour are therefore based on this slightly smaller subsample of 150. There were no missing values in the completed surveys, but one participant included a mixture of characters and numbers in their responses to the physical activity behaviour and intentions questions at Time 1, rendering their data uninterpretable. They were therefore excluded from all analyses including these two variables.

Outlier analyses revealed some extreme outliers on the physical activity behaviour and intentions measures, most likely due to participants misunderstanding the questions. For example, one participant indicated they had undertaken a total of 360 bouts of physical activity lasting at least 15 min over the past week in Survey 2 (but only 9 in Survey 1), suggesting that, in Survey 2, they may have mistakenly indicated the total number of minutes they had engaged in each intensity of physical activity during the past week. In total, seven outliers exceeded the *z* > 3.29 cut‐off (Tabachnick & Fidell, 2019) across the physical activity behaviour and intentions measures and two exceeded it for physical activity change (which we calculated by subtracting participants' Survey 1 score from their Survey 2 score). Because many of these outliers were substantial (up to 11.1 *SDs* above the mean) and we could not confidently discern the specific error each participant had made (if they had made an error), we followed the conservative approach used in previous research with this measure (Homan & Tylka, 2014) and excluded all statistical outliers from the relevant analyses. Scores for the physical activity interest variable ranged from 2 to 1020 s. There were two outliers representing participants who had spent a large amount of time engaging with the physical activity information. However, both scores were plausible (i.e. well below the maximum time we estimated participants could spend actively engaging with the information; see Measures), and we therefore retained both scores in the analyses. There was also some evidence of non‐normality for the physical activity interest variable, with skewness and kurtosis values exceeding the ±2 and ±7 cut‐offs (Byrne, 2016). As a robustness check, we repeated the analyses for this variable after conducting a log(10) transformation to bring the skewness and kurtosis values within the recommended acceptable range. The significance of the effects were unchanged. To aid interpretability, results below are based on analyses using the untransformed version of this variable.

A one‐way ANOVA on the Survey 1 physical activity scores indicated that there were no significant differences in baseline physical activity across conditions, *F*(2, 144) = 1.36, *p* = .261, *η*_*p*_^2^ = .02. One‐way ANOVAs also indicated that, across conditions, there were no significant differences in participants' social identification with the norm reference group (*F*(2, 159) = 1.50, *p* = .226, *η*_*p*_^2^ = .019) or age (*F*(2, 159) = 1.23, *p* = .294, *η*_*p*_^2^ = .015). A Chi‐square test of independence (excluding the participant who preferred not to disclose their gender, because their inclusion violated the assumptions of the test) indicated that gender distribution did not differ significantly across the experimental conditions, *χ* ^2^(2, *N* = 161) = 0.34, *p* = .85. Means and standard deviations for all variables across experimental conditions are shown in Table 3.

**TABLE 3:** Descriptive statistics for the study variables across experimental conditions and surveys in Study 2.

Variable	Control condition		Static condition		Dynamic condition	
	*M*	*SD*	*M*	*SD*	*M*	*SD*
Time 1 physical activity behaviour	35.56	23.62	32.35	23.56	41.72	33.92
Time 2 physical activity behaviour	33.65	21.85	37.74	28.12	37.45	32.75
Physical activity intentions	38.85	25.34	41.85	32.65	49.64	38.15
Physical activity interest	27.28	75.82	39.01	135.78	49.99	145.18
Self‐efficacy	3.04	0.77	3.16	0.70	3.33	0.65
Fear of pain	2.37	0.44	2.23	0.41	2.22	0.45
Social identification	4.38	0.97	4.20	1.03	4.53	0.94

### Main analyses

#### Hypotheses 1a–1c: The effect of norm condition on physical activity outcomes

To test the effect of experimental condition on physical activity intentions (H1a) and interest (H1b), we conducted one‐way between‐groups ANOVAs and to test the effect of experimental condition on physical activity behaviour, we conducted a mixed model ANOVA with time (Survey 1–Survey 2) as the within‐subjects factor and experimental condition (control–static norm–dynamic norm) as the between‐subjects independent variable. We considered the three hypotheses distinct and thus did not apply a Bonferroni correction (see Rubin, 2021) but note that the significance of the focal effects would not have changed with this correction applied. There were no significant differences in physical activity intentions (*F*(2, 156) = 1.57, *p* = .211, *η*_*p*_^2^ = .02) nor interest (*F*(2, 159) = 0.46, *p* = .631, *η*_*p*_^2^ = .006) across the three conditions. In the physical activity behaviour model, the main effect for time was not significant, *F*(1, 142) = 0.39, *p* = .84, *η*_*p*_^2^ < .001. However, there was a significant time × condition interaction, *F*(2, 142) = 4.61, *p* = .012, *η*_*p*_^2^ = .06. Planned post hoc pairwise comparisons with Tukey's (1949) correction revealed a significant difference between the static and dynamic conditions (*t*(91) = 2.91, *p* = .012), such that participants in the static condition improved their physical activity participation, whereas those in the dynamic condition demonstrated (non‐significant) reductions. There was no significant difference in terms of changes between those in the static and control conditions (*t*(96) = 2.25, *p* = .067) nor those in the dynamic and control conditions (*t*(97) = −.074, *p* = .743). Simple slope analyses demonstrated that the change in physical activity over time was significant in the static condition (*p* = .024) but not in the dynamic condition (*p* = .069) nor the control condition (*p* = .393).

#### Hypotheses 2 and 3: The mediating role of self‐efficacy and fear of pain while exercising

To assess the hypothesised mediation effects, we used the same approach as Study 1. That is, we tested three parallel mediation models (one for each dependent variable) in PROCESS (Model 4; Hayes, 2017), which assessed the indirect effects of experimental condition on the three physical activity outcomes through participants' self‐efficacy (H2) and fear of pain while exercising (H3). We again used bias‐corrected bootstrapping with 5000 resamples to calculate 95% confidence intervals for the indirect effects and coded the vectors identically to Study 1, such that the models again estimated the *relative* indirect effects of both the static and dynamic conditions compared with the control condition on the outcomes via the mediators. A change score—calculated by subtracting participants' Survey 1 scores from their Survey 2 scores—was again used as the dependent variable for the outcome we assessed twice (physical activity behaviour).

Full results from the two mediation models are shown in Table 4. Providing partial support for H2, there was some evidence of mediation through self‐efficacy. Specifically, being in the dynamic condition compared with the control condition predicted greater physical activity intentions via greater self‐efficacy. H3 was not supported, with all indirect effects via fear of pain while exercising non‐significant.^4^

**TABLE 4:** Full results of the mediation models testing Hypotheses 2 and 3 in Study 2.

Outcome	Comparison	Mediators										Direct effects		Total effects	
		Self‐efficacy					Fear of pain while exercising								
		Condition → mediator		Mediator → outcome		Indirect effect	Condition → mediator		Mediator → outcome		Indirect effect *β* [95% CI]				
		*β*	*p*	*β*	*p*	*β* [95% CI]	*β*	*p*	*β*	*p*		*β*	*p*	*β*	*p*
PA behaviour	Control vs. static	.06	.75	.12	.20	.01 [−.06, .08]	−.24	.24	.19	.04	−.05 [−.18, .02]	.48	.02	.44	.03
	Control vs. dynamic	.37	.07	.12	.20	.05 [−.01, .15]	−.32	.11	.19	.04	−.06 [−.19, .01]	−.13	.52	−.14	.46
PA intentions	Control vs. static	.13	.51	.43	<.001	.06 [−.11, .22]	−.30	.13	−.10	.22	.03 [−.02, .11]	.01	.97	.09	.64
	Control vs. dynamic	.42	.03	.43	<.001	.18 [.02, .34]	−.35	.07	−.10	.22	.03 [−.02, .12]	.11	.51	.33	.09
PA interest	Control vs. static	.17	.38	−.004	.96	<−.001 [−.04, .03]	−.32	.09	.10	.27	−.03 [−.10, .01]	.13	.51	.10	.34
	Control vs. dynamic	.41	.03	−.004	.96	−.002 [−.07, .05]	−.34	.08	.10	.27	−.04 [−.11, .01]	.22	.26	.19	.34

## DISCUSSION

In Study 2, we sought to gain a broader understanding of how static and dynamic norm messages impact physical activity outcomes by testing their effects on people's physical activity‐related cognitions and behaviours during daily life. There was only partial support for our hypotheses. However, the effects we observed were consistent with Study 1. First, the static norm messages were the only messages that directly influenced participants' behaviours. Findings therefore provide further support for the preferential use of such messages in physical activity contexts and the first evidence that they might be an effective way to improve older adults' physical activity behaviours.

Also similar to Study 1, there was some evidence that the dynamic norm messages affected the physical activity outcomes indirectly—in this instance predicting greater physical activity intentions via a positive impact on self‐efficacy. This suggests that messages indicating similar aged others have improved their behaviours and gained confidence in their ability to be active can enhance older adults' confidence that they too can be active, with downstream benefits for their intentions to do so. Indeed, self‐efficacy was also a positive predictor of physical activity behaviour, but this effect was smaller and non‐significant. We found no significant indirect effects through fear of pain while exercising. Receiving norm messages of either type compared with no message tended to predict lower fear of pain while exercising, but the effects were non‐significant, and fear of pain was not a significant negative predictor of the physical activity outcomes. One possible reason for these non‐significant indirect effects through fear of pain while exercising is that there might have been an absence of pain, disability or mobility issues in our sample. This may have meant that participants' fear of pain while exercising was already low, thus limiting the capacity for the norm manipulations to reduce this.

Given the mediation findings, intervention designers might still consider using norm messages to help boost people's self‐efficacy and reduce their fear of pain while exercising. However, recognising that these messages may have moderate effects, they may wish to use them as part of a broader intervention—for example, alongside existing evidence‐based approaches, such as motivational interviewing for building self‐efficacy (O'Halloran et al., 2016) and pain neuroscience education for reducing fear of pain while exercising (Louw & Riera‐Gilley, 2024).

Key strengths of Study 2 included the experimental design, novel population for physical activity norms research and multiple physical activity outcomes it included. However, although we assessed physical activity interest with a behavioural indicator, unlike in Study 1, we were unable to measure physical activity behaviours objectively. Results should therefore be interpreted with appropriate caution given the discrepancies that can exist between people's self‐reported and objectively assessed physical activity behaviours (Steene‐Johannessen et al., 2016). Further research assessing the effects of both types of norm messages on objective measures of people's physical activity behaviours during daily life (e.g. obtained via accelerometers) would be valuable.

## GENERAL DISCUSSION

Across two studies, we examined the relative effects of static and dynamic norm messages on a range of physical activity outcomes. Overall, our findings suggest that static norm messages may be a more effective way to improve people's physical activity behaviours. There was also some evidence that enhanced motivation and self‐efficacy are two pathways through which norm messages can have positive effects. Findings have both theoretical and applied implications.

First, our findings extend existing understanding regarding the most effective types of norm messages for changing physical activity behaviours. Dynamic messages have often been shown to be more effective than static messages for changing other behaviours (e.g. Sparkman & Walton, 2017). However, our findings suggest that static messages may be more effective for changing physical activity behaviours. One possible reason for this is that there is something specific about physical activity that makes static norm messages particularly effective in these contexts. For example, they may invoke a feeling of competitiveness that static messages related to other behaviours do not. That is, people may feel a stronger desire to match or outperform others on exercise tasks or in relation to their overall physical activity participation than they do, for example, to save more water than others while doing laundry (see Sparkman & Walton, 2017, Study 5). At the same time, informing people that others have changed their physical activity behaviours via a dynamic norm message without providing the underlying norm itself (e.g. the ‘average’ score on an exercise task or current proportion of people who are exercising regularly) may not invoke the same competitiveness (and thus have as strong an effect) because it does not give them either a concrete ‘target’ to aim for or an understanding of the proportion of others that are outdoing them.

Along these lines, it is also notable that our studies provided relatively stringent tests of the effects. In Study 1, participants were explicitly instructed to travel as far as they could on both trials, limiting the potential for improvements. In Study 2, we aimed to change people's physical activity behaviours during daily life, which is often difficult to achieve, particularly with limited contact with participants (Vandelanotte et al., 2007). We also focused on older adults—a population who often experience greater barriers (e.g. mobility limitations) to engaging in additional physical activity, even if they have sufficient motivation (Kilgour et al., 2024). Thus, although further research is needed to better understand *why* static norm messages appear to be more effective in physical activity contexts (e.g. if it is because they invoke greater competitiveness), it is particularly promising that they improved participants' behaviours given the nature of our studies. Our research therefore provides further support for their use as part of physical activity interventions.

At the same time, when interpreting the results, two features of our studies are important to note that may have contributed to the observed effects. First, we made participants' social identity as a member of the norm reference group salient prior to presenting the norm messages, given evidence that this can enhance their effectiveness (e.g. see Liu et al., 2019). Second, we supplemented our norm messages with additional information. Specifically, in Study 1, we sought to contextualise the messages by providing participants feedback regarding how *their* Trial 1 score compared with the norm (static condition) or the score *they* would achieve if they followed the pattern shown by others (dynamic condition). In Study 2, we provided norm information related to our hypothesised mediators (as well as physical activity), and participants also received information about the benefits of physical activity and how to incorporate it into their daily lives as part of our physical activity interest measure (although they spent less than 1 min engaging with this information on average). These manipulation features were experienced by all participants, likely minimising their impact on *relative* differences in the outcomes across conditions. Indeed, it is possible that making all participants' social identity salient may have attenuated the differences between conditions because this might have activated group‐based motivations to engage in positive physical activity behaviours among participants in the control condition (see also Liddelow et al., 2025). However, it is possible that these features may have enhanced the improvements we observed *within* the static conditions in the two studies. Indeed, dynamic norms may have been relatively more effective in past research because researchers have typically not incorporated these additional features and instead compared their impact as standalone messages. From an applied perspective, we would therefore recommend that, where feasible, intervention designers attempt to make the norm referent group salient and incorporate accompanying feedback and information elements when using static norm‐based interventions. Given the limited time our participants tended to spend engaging with the information we included, researchers may, however, wish to provide this information in a different medium where practical (e.g. deliver it in person) or attempt to find ways to encourage greater engagement with it if it needs to be provided via webpages (e.g. include interactive elements).

### Limitations and future research

This research had several strengths. Notably, the two studies tested the hypothesised effects experimentally in different (lab and real‐world) contexts, on a range of physical activity outcomes, and with diverse samples. Nevertheless, some limitations and avenues for future research should be noted. First, like most physical activity norms research (see Kim et al., 2019, for a review) and research comparing both types of norm message (e.g. see Sparkman & Walton, 2017), our studies examined only the short‐term effects of the messages. Further research is therefore required to assess whether either or both types of messages can be effective in creating sustained behaviour change, perhaps as part of an intervention in which they are communicated on multiple occasions via different sources (e.g. email, social media and posters). Along these lines, it is possible that behaviours such as physical activity that are continual and effortful in nature and desirable to increase benefit more from repeated exposure to norm messages or feedback than ‘one‐off’ behaviours (e.g. getting a vaccine) and/or those that it is desirable to reduce (e.g. meat eating) do. Further research testing the relative effects of static and dynamic norm messages on different behaviours could enhance understanding regarding how these different characteristics of behaviours shape their amenability to normative influence and the type and extent of norm messaging that is most beneficial to influence them.

As alluded to above, further research examining the mechanisms through which norm messages influence physical activity (and other) behaviours would also be valuable. Here, researchers could consider both feelings that specific messages may trigger (e.g. competitiveness from physical activity‐related messages; see above) and key barriers to the behaviour that norm messages could help address. Sparkman and Walton (2019) found that dynamic norm messages tended to exert effects via their impact on such barriers (e.g. perceived ability to change for smoking cessation). Notably though, the researchers' norm messages remained focused on changing normative perceptions related to the behaviour. Researchers could also consider designing norm messages that seek to change normative perceptions related to the barriers (as we attempted to do in relation to self‐efficacy and fear of pain in Study 2). For example, recognising that lack of time is a commonly cited barrier to physical activity (Sequeira et al., 2011), researchers could use messages that highlight that a high or increasing proportion of people are waking up early to exercise and then assess whether any beneficial effects on behaviour are underpinned by an increase in people's perception that it is feasible to fit exercise into their daily regime.

## CONCLUSION

Across two studies, we found that static norm messages, combined with feedback (Study 1) and information (Study 2), were more effective for improving people's physical activity behaviours than dynamic norm messages combined with feedback and information. We also found some evidence that both types of messages can have positive effects on physical activity outcomes by boosting people's motivation and self‐efficacy but observed these indirect effects less consistently. Using feedback indicating that positive physical activity behaviours are typical could be a fruitful way to improve people's behaviours.

## CONFLICT OF INTEREST STATEMENT

The authors declare no conflicts of interest.

## ETHICS STATEMENT

Ethics approval for the two studies included in the manuscript was obtained from the Human Research Ethics Committee at the first author's institution (#2024/547 and #2025/0112).

## Supporting information


**Data S1.** The norm messages shown to participants in the static and dynamic conditions in Study 2.

**Data S2.** The page shown to participants at the end of Survey 1 in Study 2. Time spent engaging with this material provided our measure of physical activity interest.

**Table S1.** Results from mediation models testing the relative effects of being in the dynamic versus static condition in Study 1.

**Table S2.** Results from mediation models testing the relative effects of being in the dynamic versus static condition in Study 2.

## Data Availability

The data that support the findings of this study are available from the corresponding author upon reasonable request.

## ENDNOTES

1 While noting that a longer rest interval or splitting participants' two trials across separate days would have enabled more complete recovery, we opted for a 5‐min interval because this helped (a) ensure the feasibility of data collection and (b) minimise the impact of possible confounds on participants' relative scores across the two trials (e.g. if they had completed trials on separate days and factors such as how well they had eaten or slept before these may have impacted their relative performance).

2 Although it was not necessary to make participants' social identity salient in the control condition, this was done to maximise consistency in the protocol across conditions.

3 We also tested the mediation models with the dynamic condition set as the reference condition to prompt PROCESS to provide the relative effects of being in this compared to the static condition. No indirect effects were significant for this comparison. Full results are provided in Table S1.

4 As in Study 1, we also tested the mediation models with the dynamic condition set as the reference condition to prompt PROCESS to provide the relative effects of being in this compared to the static condition. No indirect effects were significant for this comparison. Full results are provided in Table S2.
