# Supplementary material for: The effect of dynamic and static norm messages on physical activity behaviours: Evidence from two experimental studies
Source: Appl Psychol Health Well Being. 2026 Aug 19;18(4):e70209. doi: 10.1111/aphw.70209 (PMC13487498; doi:10.1111/aphw.70209)
Supplement: Supplementary file 1 [file APHW-18-0-s001.docx]

**Supplementary materials 2.** The page shown to participants at the end of Survey 1 in Study 2. Time spent engaging with this material provided our measure of physical activity interest.

Thank you for taking part in our survey. We will invite you to complete the second and final survey in this study in a week’s time and would be very grateful if you could do this.

In the meantime, if you are interested in learning more and improving your own healthy lifestyle by increasing the amount of physical activity you do, there is some information to help you do so on this page, just click on the headings below if you want to read more. When you are finished reading, you can click ‘next’ at the bottom of the page to finish the survey.

Thank you again for your participation.

**What are the benefits of being physically active?**

Physical activity is good for us in lots of ways.

First of all, it helps protect against a whole host of physical health problems and diseases, including:

- Heart disease
- Stroke
- Certain types of cancers
- Type 2 diabetes
- Osteoporosis

It has also been shown to help our mental health and wellbeing. For example, it can help:

- Reduce anxiety
- Buffer against stress
- Buffer against and reduce the symptoms of depression
- Improve mood
- Improve and maintain cognitive functioning

You can learn more about what happens in the body when you exercise in the video below.

<https://www.youtube.com/watch?v=wWGulLAa0O0>

**How much physical activity should I do?**

The NHS recommends that older adults aged 65+ should do some kind of physical activity every day. More specifically, they also recommend:

- Reducing the amount of time spent sitting or lying down and breaking up long periods of not moving with some activity.
- Doing at least 150 minutes of moderate intensity activity a week, or 75 minutes of vigorous intensity activity per week, or some combination of the two.

Moderate intensity activity is activity that raises your heart rate, and makes you breathe faster and feel warmer. One way to tell that you’re working at a moderate intensity level is if you can still talk, but not sing!

Vigorous intensity activity makes you breathe hard and fast. If you’re working at this level, then you will not be able to say more than a few words without pausing for breath.

To get started, you could try this 10-minute living room workout:

<https://youtu.be/O5YX5xg8Seg?si=wSuULeZT_T4DxUZn>

The NHS also recommends:

- Doing activities that improve strength, balance, and flexibility on at least 2 days a week.

Not sure how to do this? Check out these two videos about increasing your strength at home:

<https://youtu.be/NkHMLUmckUI?si=X8eIjglqRoOEzeIn>

<https://youtu.be/G_o4CnisSe4?si=umYB3IV4D-3RIzui>

**“I want to get more active, what could I do?”**

Being more active doesn't have to mean working up a sweat or joining a gym. It can mean just moving more each day in whatever way works for you. Being more active shouldn't feel like a chore, so it's important to find something you enjoy.

Getting active can seem daunting if you don’t currently move much – so if that’s you, then start small. It’s always a good idea to build up your activity levels gradually. Lots of everyday activities count as exercise, so look for simple ways you can begin to move more that fit in with your daily routine.

You could start by:

- getting up to make a cup of tea and stretching while the kettle boils
- walking to the shops instead of driving or choosing a parking space a bit further away from the shops if you drive there
- using stairs instead of lifts or escalators
- doing more housework or gardening

Once you’re going, or if you’re already quite active, then there are lots of other things you could try. For example, you could:

- Go for a walk with a friend instead of having a cup of tea together
- Join a dance class at the local town hall
- Go swimming, bike riding, or hiking
- Join a local walking football or netball club

In fact, walking football and walking netball are both very common ways older adults in the UK are getting active! It is estimated that more than 60,000 people in England are playing walking football, and more than 30,000 people in England are playing walking netball!

You can watch these videos to see if a walking sport is for you:

<https://www.youtube.com/watch?v=h2ndpPW_u-A>

<https://youtu.be/qzQfnv7sFPg?si=wVzv-vu6ay4IXPLY>

<https://youtu.be/wMh9mreDvJA?si=oPe2c53y-hJ68hg6>
