# Supplementary material for: The effect of dynamic and static norm messages on physical activity behaviours: Evidence from two experimental studies
Source: Appl Psychol Health Well Being. 2026 Aug 19;18(4):e70209. doi: 10.1111/aphw.70209 (PMC13487498; doi:10.1111/aphw.70209)
Supplement: Supplementary file 2 [file APHW-18-0-s002.docx]

**Supplementary materials 1.** The norm messages shown to participants in the static and dynamic conditions in Study 2.

**Static Condition**

As part of this study, we have reviewed the latest research and found that the evidence shows **a lot of older adults are being active**! Specifically, one study found that **79% of older adults** over 65 are **exercising regularly**.

Older adults can lack confidence when it comes to being physically active due to concerns about safety and injury. However, one of the studies we reviewed found that **3 in 4 older adults** aged 65 and over **are confident** when it comes to being active.

Proportion of Older Adults who are Confident in their Ability to be Physically Active

|  |  |
| --- | --- |
| Confident | 3 |
| Not Confident | 1 |

We know that some older adults believe that moving their body will cause them pain. But did you know that **exercise can be a natural painkiller**?

In our research, we found a study which showed that the number of older adults using exercise for pain relief is **82%.**

**Dynamic condition**

As part of this study, we have reviewed the latest research and found that the evidence shows **more and more older adults are getting active**! One study found that **79% of older adults** aged over 65 were exercising **more regularly** than they did when they were 55.

Average Physical Activity Levels in a Sample of Adults Over Time

|  |  |
| --- | --- |
| 55 years | 2 |
| 65+ years | 5.5 |

Older adults *used* to lack confidence when it came to being physically active due to concerns about safety and injury. However, **this is changing**. One of the studies we reviewed found that **3 in 4 older adults** aged 65 and over **have become more confident in the last 5 years** when it comes to being active.

Change in Adults Confidence in Ability to be Physically Active

|  |  |
| --- | --- |
| 1 | 4 |
| 2 | 40 |

We know that some older adults believe that moving their body will cause them pain. But did you know that **exercise can be a natural painkiller**?

In our research, we found a study which showed that, **in the last 5 years**, the number of older adults using exercise for pain relief has **increased by 82%.**

Number of Older Adults Exercising Regularly for Pain Relief

|  |  |
| --- | --- |
| 1 | 10 |
| 2 | 38 |
