# Supplementary material for: The effect of dynamic and static norm messages on physical activity behaviours: Evidence from two experimental studies
Source: Appl Psychol Health Well Being. 2026 Aug 19;18(4):e70209. doi: 10.1111/aphw.70209 (PMC13487498; doi:10.1111/aphw.70209)
Supplement: Supplementary file 3 [file APHW-18-0-s003.docx]

**Supplementary table 2.** Results from mediation models testing the relative effects of being in the dynamic versus static condition in Study 2.

|  |  |  |  | Mediators | | | | | | | | |  | Direct effects | |  | Total effects | | | |
| --- | --- | --- | --- | --- | --- | --- | --- | --- | --- | --- | --- | --- | --- | --- | --- | --- | --- | --- | --- | --- |
|  |  |  |  | Self-efficacy | | |  | Fear of pain | | | | |  |  |  |  |  | |  |  |
|  |  | Condition →  Mediator | | Mediator →  Outcome | | Indirect effect |  | Condition →  Mediator | | Mediator →  Outcome | | Indirect effect |  |  |  |  | |  | | |
| Outcome | Comparison | β | *p* | β | *p* | β [95% CI] |  | β | *p* | β | *p* | β [95% CI] |  | β | *p* | β | | *p* | | |
| PA behaviour | Dynamic vs. Static | -.30 | .14 | .12 | .20 | .04 [-.14, .01] |  | .08 | .70 | .19 | .04 | .02 [-.09, .10] |  | .61 | .003 | .59 | | .004 | | |
| PA intentions | Dynamic vs. Static | -.29 | .13 | .43 | <.001 | -.13 [-.29, .07] |  | .06 | .77 | -.10 | .22 | -.01 [-.06, .05] |  | -.11 | .53 | -.24 | | .22 | | |
| PA interest | Dynamic vs. Static | -.24 | .21 | -.004 | .96 | .001 [-.04, .04] |  | .02 | .94 | .10 | .27 | .002 [-.04, .05] |  | -.09 | .64 | -.09 | | .64 | | |
