# Supplementary material for: The effect of dynamic and static norm messages on physical activity behaviours: Evidence from two experimental studies
Source: Appl Psychol Health Well Being. 2026 Aug 19;18(4):e70209. doi: 10.1111/aphw.70209 (PMC13487498; doi:10.1111/aphw.70209)
Supplement: Supplementary file 4 [file APHW-18-0-s004.docx]

**Supplementary table 1.** Results from mediation models testing the relative effects of being in the dynamic versus static condition in Study 1.

|  |  |  |  | Mediators | | | | | | | | |  | Direct effects | |  | | Total effects | | |
| --- | --- | --- | --- | --- | --- | --- | --- | --- | --- | --- | --- | --- | --- | --- | --- | --- | --- | --- | --- | --- |
|  |  |  |  | Task motivation (change) | | |  | Self-efficacy | | | | |  |  |  | |  | |  |  |
|  |  | Condition →  Mediator | | Mediator →  Outcome | | Indirect effect |  | Condition →  Mediator | | Mediator →  Outcome | | Indirect effect |  |  |  | |  | |  |  |
| Outcome | Comparison | β | *p* | β | *p* | β [95% CI] |  | β | *p* | β | *p* | β [95% CI] |  | β | *p* | |  | | β | *p* |
| Performance | Dynamic vs. Static | 0.02 | .94 | 0.26 | .002 | 0.005 [-0.12, 0.15] |  | 0.14 | .57 | 0.50 | <.001 | 0.07 [-0.15, 0.29] |  | 0.30 | .10 | |  | | 0.38 | .11 |
| Maximum heart rate | Dynamic vs. Static | 0.02 | .94 | 0.23 | .026 | 0.004 [-0.12, 0.12] |  | 0.14 | .57 | 0.32 | .001 | 0.05 [-.12, 0.26] |  | .06 | .79 | |  | | 0.11 | .65 |
| Average heart rate | Dynamic vs. Static | 0.02 | .94 | 0.16 | .15 | 0.003 [-0.08, 0.21] |  | 0.14 | .57 | 0.26 | .001 | 0.04 [-0.09, 0.21] |  | -0.06 | .78 | |  | | -.02 | .92 |
